# Extensive Evaluation of Morphological Statistical Harmonization for Brain Age Prediction

**DOI:** 10.3390/brainsci10060364

**Published:** 2020-06-11

**Authors:** Angela Lombardi, Nicola Amoroso, Domenico Diacono, Alfonso Monaco, Sabina Tangaro, Roberto Bellotti

**Affiliations:** 1 Istituto Nazionale di Fisica Nucleare, Sezione di Bari, 70125 Bari, Italy; angela.lombardi@ba.infn.it (A.L.); nicola.amoroso@ba.infn.it (N.A.); domenico.diacono@ba.infn.it (D.D.); alfonso.monaco@ba.infn.it (A.M.); roberto.bellotti@ba.infn.it (R.B.); 2 Dipartimento di Scienze del Farmaco, Università degli Studi di Bari ’Aldo Moro’, 70125 Bari, Italy; 3 Dipartimento di Scienze del Suolo, della Pianta e degli Alimenti, Università degli Studi di Bari ’Aldo Moro’, 70125 Bari, Italy; 4 Dipartimento Interateneo di Fisica, Università degli Studi di Bari ’Aldo Moro’, 70125 Bari, Italy

**Keywords:** aging, neurodevelopment, age prediction, multi-site harmonization, morphological analysis, FreeSurfer

## Abstract

Characterizing both neurodevelopmental and aging brain structural trajectories is important for understanding normal biological processes and atypical patterns that are related to pathological phenomena. Initiatives to share open access morphological data contributed significantly to the advance in brain structure characterization. Indeed, such initiatives allow large brain morphology multi-site datasets to be shared, which increases the statistical sensitivity of the outcomes. However, using neuroimaging data from multi-site studies requires harmonizing data across the site to avoid bias. In this work we evaluated three different harmonization techniques on the Autism Brain Imaging Data Exchange (ABIDE) dataset for age prediction analysis in two groups of subjects (i.e., controls and autism spectrum disorder). We extracted the morphological features from T1-weighted images of a mixed cohort of 654 subjects acquired from 17 sites to predict the biological age of the subjects using three machine learning regression models. A machine learning framework was developed to quantify the effects of the different harmonization strategies on the final performance of the models and on the set of morphological features that are relevant to the age prediction problem in both the presence and absence of pathology. The results show that, even if two harmonization strategies exhibit similar accuracy of predictive models, a greater mismatch occurs between the sets of most age-related predictive regions for the Autism Spectrum Disorder (ASD) subjects. Thus, we propose to use a stability index to extract meaningful features for a robust clinical validation of the outcomes of multiple harmonization strategies.

## 1. Introduction

The structure of human brain changes throughout the lifespan, giving rise to different processes with typical patterns in specific time intervals [1]. Brain maturation occurs from childhood to young adulthood, with trajectories involving heterogeneous variability of anatomical features in different cortical regions. Both spatial and temporal patterns of cortical descriptors, white and gray matter volume, have been associated with maturation trajectories [2,3,4]. Several studies described variable decreases and increases of cortical thickness reflecting changes of tissue microstructure [5,6,7]. In addition, increases in white matter volume and continuing myelination have been observed during neurodevelopment [8]. Brain aging is affected by progressive atrophy patterns and cortical thickness decline in elderly lifespan [9,10]. Characterizing both neurodevelopmental and aging brain structural trajectories is important for understanding normal biological processes and atypical patterns that are related to pathological phenomena. As an example, accelerated aging atrophy has been found in neurodegenerative diseases, such as Alzheimer’s Diseases [11,12]; other studies have investigated altered morphological patterns during neurodevelopment in neurological disorders, such as Autism Spectrum Disorder (ASD) [13,14]. Recently, a comprehensive index has been introduced to describe the brain age of a subject [15]. The main rationale of this approach is to develop robust chronological age prediction models that allow for estimating both the maturation and aging trajectories of the human brain using a population dataset spanning a range of ages. Accordingly, the age of a subject can be predicted and the deviation (i.e., delta) between the brain age and the chronological age can be assessed in order to derive information regarding the brain health status. Indeed, positive delta scores have been related to protective or resilience factors and negative delta scores have been linked to pathological conditions and neurological and neurodevelopmental disorders [16,17,18].

Brain age prediction models have been improved by the joint application of large datasets and advanced machine learning algorithms. By using magnetic resonance imaging (MRI) features and multivariate analyses, several biomarkers have been identified to accurately describe both neurodevelopment and aging processes [19,20,21,22,23,24]. The main steps of these approaches include: feature extraction, feature selection, and fitting the regression model to the biological age. According to these several state-of-the-art studies, machine learning regression algorithms can overcome common problems such as handling correlated predictors, redundancy and model overfitting. Moreover, machine learning approaches could take into account also nonlinear interactions among predictors and between the predictors and the outcome variable, without assuming any regression formula. Among structural features, morphological descriptors such as cortical thickness, surface area, mean curvature, white matter (WM) and gray matter (GM) volumes have been proved to be reliable age-related biomarkers, reporting mean absolute error 4 years [19,24,25,26,27].

Initiatives to share open access morphological data contributed significantly to the advance in brain structure characterization. Indeed, such initiatives allow large brain morphology cross-sectional datasets to be shared, which increases the statistical sensitivity of the outcomes and promotes the development of reproducible research methods [28]. However, using neuroimaging data from multi-site studies requires to “harmonize” data across sites: scanner effects that are related to hardware and protocols can influence brain morphology estimates acting as “batch effects”. Recently, ComBat [29], a batch-effect correction tool used in genomics, has been adapted for harmonizing cortical thickness measurements obtained from multiple sites [30] and multi-site DTI studies [31]. ComBat was found to be an effective harmonization technique that both removes unwanted variation that is associated with site and preserves biological associations in the data. In their work, Fortin et al. showed the effectiveness of ComBat algorithm to remove site effects, while preserving the biological variability that is associated with the age of the subjects of four large multi-site datasets. In particular, the authors evaluated the effects of harmonization on the prediction of age by using the harmonized cortical thickness as features with several regression algorithms. They showed that ComBat significantly improved both the average prediction accuracy when compared with the raw data and the correlation between the imaging outcome and the factor of interest (i.e., age).

The aim of this work is to compare different harmonization strategies to investigate the effect of statistical harmonization on a multi-site neuroimaging dataset. In particular, the Autism Brain Imaging Data Exchange (ABIDE) dataset was adopted in order to extract morphological features and predict the biological age of both control and ASD subjects by using machine learning regression models. We provide a robust framework to compare the performance of the machine learning models and quantify the stability of the most relevant anatomical features. The performance of the models and the most significant morphological features were analyzed to: (i) verify whether different harmonization techniques influence the accuracy of predictive models; and, (ii) investigate the effects of the harmonization on the anatomical regions with the most significant impact on age prediction for both groups of subjects.

## 2. Materials and Methods

### 2.1. Subjects

The Autism Brain Imaging Data Exchange (ABIDE) cohort includes imaging data from 1112 participants. ABIDE represents a consortium effort to aggregate MRI datasets from individuals with autism spectrum disorder and age-matched typically-developing controls (NC) [32]. ABIDE was conceptualized to identify ASD-related factors that can vary across studies and to guide future efforts to increase harmonization among research groups. The contributions per site ranged from 13 to 79 participants with ASD and 13 to 105 NC. The dataset includes three Tesla, T1-weighted MRI acquired from 17 sites; images and acquisition details are available at http://fcon_1000.projects.nitrc.org/indi/abide. Initial contributions were sought from members of the ADHD-200 Consortium conducting autism research (Kennedy Krieger Institute, NYU Langone Medical Center, Oregon Health Science University, University of Pittsburgh). Invitations to participate were extended based on personal communications, recent publications, and conference presentations. All of the investigators willing and able to openly share previously collected MRI data from individuals with ASD and age- and sex-group matched TC were included. Institutional Review Board (IRB) approval to participate, or explicit waiver to provide fully anonymized data, was required prior to data contribution. All of the participating sites received local Institutional Review Board approval for acquisition of the contributed data. We only considered male subjects due to the high imbalance between male and female subjects in the data sample. For quality assessment, we firstly evaluated a comprehensive index of anatomical quality metrics by computing the principal component analysis of the contrast to Noise Ratio, percent of artifact voxels, and signal to noise ratio. Subsequently, we used the median absolute deviation (MAD) criterion [33] to exclude the extreme outliers of the anatomical index. From the remaining T1 images, we selected subjects with age 40 obtaining a final sample of N=654 from 17 sites, including N1=374 typically-developing participants, mean age = 17.25 years, std age = 6.89, age range = [6.47−39.39]; N2=280 ASD subjects, mean age = 16.62 years, std age = 6.32, and age range = [7.15−39.10].

### 2.2. Morphological Features

The software tool FreeSurfer (https://surfer.nmr.mgh.harvard.edu/) was used to extract the morphometric properties of both cortical and sub-cortical brain structures. In particular, the recon-all pipeline of FreeSurfer v.5.3.0 [34,35,36] was used to extract brain morphological statistical features. Recon-all is a fully automated workflow that performs all of the FreeSurfer cortical reconstruction and sub-cortical segmentation steps in a unified pipeline. It includes several processing stages, such as motion correction, non-uniform intensity normalization, Talairach transform computation, intensity normalization, skull stripping, sub-cortical segmentation, and cortical parcellation steps. More details regarding the steps of recon-all workflow can be found at https://surfer.nmr.mgh.harvard.edu/fswiki/recon-all. The Desikan-Killiany atlas [37] was used to perform the cortical segmentation of MRI into 68 regions of interest. The sub-cortical segmentation into 40 regions of interest was performed by means of the Aseg Atlas [36]. After the segmentation of cortical and sub-cortical brain regions, different morphometric and intensity properties of these regions can be computed. In particular, the following statistical descriptors are automatically estimated by the recon-all pipeline:

- volume, intensity mean, standard deviation, minimum, maximum, and range of 40 sub-cortical brain structures and white matter parcellation of brain cortex;
- volume, surface area, Gaussian curvature, mean curvature, curvature index, folding index, thickness mean, and thickness standard deviation for the 34 cortical brain regions of each hemisphere; and,
- global brain metrics, including surface and volume statistics of each hemisphere; total cerebellar gray and white matter volume, brainstem volume, corpus callosum volume, and white matter hypointensities.

For the sake of clarity, we divided all the FreeSurfer features into four categories:

- global metrics (21 features);
- gortical metrics (544 features resulting from eight metrics for the 68 cortical regions of interest);
- sub-cortical metrics (240 features resulting from 6 metrics for the 40 sub-cortical regions of interest); and,
- WM metrics (408 features resulting from six metrics for the 68 regions of interest).

Each anatomical metric was divided by total intracranial volume (ICV) of the corresponding subject in order to normalize the statistical features to brain size and reduce their variance. We also performed additional analyses without correcting ICV to test the impact of this correction on harmonization and no differences were found between the two methods. Finally, the ABIDE cohort was described by two matrices $N_{1}\times P$ and $N_{2}\times P$, with $N_{1}=374$, $N_{2}=280$, and P=1213, where each row represents a single subject described with *P* morphological features.

### 2.3. Overview of the Framework

Usually, age prediction is performed while using multiple features that were extracted from one or more imaging modalities. A dataset is then defined by including multiple subjects’ features and their true ages. The dataset is fed into a supervised machine learning algorithm that learns to predict the subjects’ ages from their brain imaging features. The machine learning algorithm aims at predicting the brain age of a given subject while minimizing the deviation from the true age and avoiding overfitting. Different metrics, such as Mean Absolute Error (MAE), are commonly employed in order to evaluate the delta between the predicted age and true age of the subjects. In addition, several feature selection techniques can be applied to remove irrelevant, noisy, and redundant features, avoiding overfitting and improving prediction performance, reducing the computational complexity of the learning algorithm, and proving a deeper insight into the data, which highlights which of the features are most informative for age prediction [38,39].

In this study, a machine learning framework was developed in order to:

- compare multiple harmonization strategies;
- identify the most effective age predictive model;
- select only the most significant features among the total set of features; and,
- compare the stability of the most age-related anatomical regions of interest across harmonization strategies.

**Figure 1:**
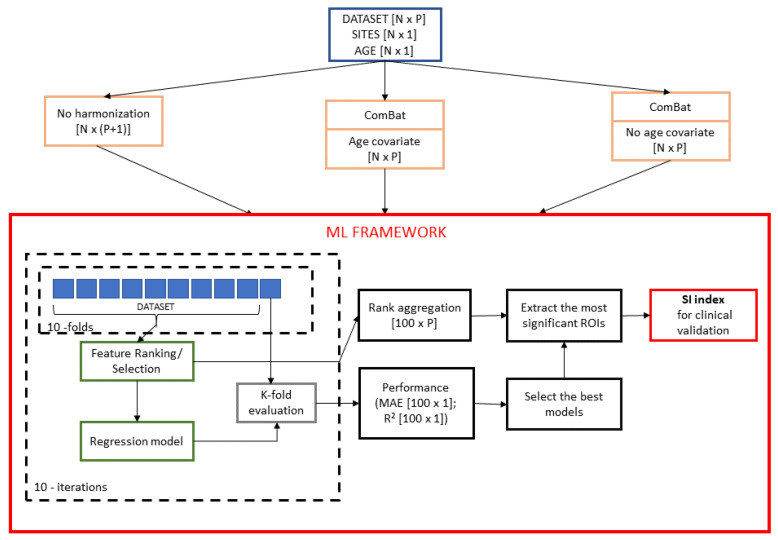
Statistical framework to compare the three harmonization strategies and select the most age-related predictive features. Three harmonization strategies were applied to each of the two dataset (i.e., NC and ASD). For each resulting dataset, a nested feature selection was performed on the training set in each round of the k-fold validation. Subsequently, 100 stepwise regression models were trained by progressive increasing the training set size. A consensus ranking procedure was used to select the most stable features with the lowest mean absolute error. This procedure was repeated for each of the three regression algorithms—SVR, RF, and Lasso—resulting in nine combinations of “harmonization strategy–regression model”. The performance of the models were compared in order to select the most effective machine learning algorithm for age prediction. For the best regression model, the sets of features that resulted from different harmonization strategies are compared using a stability index to quantify the effects of the harmonization and support clinical evaluation.

A schematic overview of the framework is shown in Figure 1. Firstly, three harmonization strategies were applied to both controls and ASD datasets. Ten re-sampling of a 10-fold cross-validation were executed, producing 100 bootstraps of each dataset. In each iteration, nine-folds of the original dataset were input to each of the three regression models (Support vector Regression, Random Forest, Lasso) and then stepwise models were trained for ranked subsets of increasing size (e.g., the top five, 15, 20, and so on up to P ranked features). Each stepwise model was tested on the left fold and the performances of each model were stored for successive evaluations. The purpose of stepwise analysis is to identify the particular subset of features that minimizes the age prediction error [40]. As a result, the output of the analysis is the number of non-redundant features $k_{opt}$ to consider in order to yield the best performance of the three models for each harmonization strategy together with a matrix of size [100×P] of ranked/selected features at each iteration. The performances of the models were compared in order to select the most effective machine learning algorithm for age prediction. Finally, for such regression model, we compared the sets of features that result from different harmonization strategies by using a stability index to quantify the effects of the harmonization. The main steps of the framework are described in the following sections in more details.

### 2.4. Statistical Harmonization

Linear regression is the most popular method for performing harmonization, adjusting the images for site effects. It does not take into account the potential confounding between the site variables and the biological covariates of interest in the study. For the regression model, the morphological measurement $y_{ij\upsilon }$ for imaging site *i*, for participant *j* and feature υ is expressed as:

(1) $$y_{ij\upsilon }=\alpha_{\upsilon }+Z_{ij}\Theta_{\upsilon }+\epsilon_{ij\upsilon ,}$$

where $\alpha_{\upsilon }$ is the average morphological metric for the reference site for feature υ; *Z* is the matrix of site indicators; $\Theta_{\upsilon }$ is the [c×1] vector of the coefficients associated with *Z* for feature υ; and, $\epsilon_{ij\upsilon }$ is the residual term. For each feature separately, regular ordinary least squares (OLS) is used to estimate the parameter vector $\Theta_{\upsilon }$. The removal of site effects is done by subtracting the estimated site effects. The adjusted residuals harmonization method supervises the removal of site effects by adjusting for biological covariates, while using the modified linear regression model:

(2) $$y_{ij\upsilon }=\alpha_{\upsilon }+W_{ij}\beta_{\upsilon }+Z_{ij}\Theta_{\upsilon }+\epsilon_{ij\upsilon },$$

where *W* is the matrix of biological covariate of interests; $\beta_{\upsilon }$ is the [c×1] vector of the coefficients that are associated with $W_{ij}$ for feature υ.

The ComBat harmonization model extends the adjusted residuals harmonization model presented in Equation (2) and assumes that the expected values of the imaging feature measurements can be modeled as a linear combination of the biological variables and the site effects, whose error term is modulated by additional site-specific scaling factors [30]. ComBat models the morphological measurement $y_{ij\upsilon }$ for imaging site *i*, for participant *j* and feature υ as:

(3) $$y_{ij\upsilon }=\alpha_{\upsilon }+W_{ij}\beta_{\upsilon }+\gamma_{i\upsilon }+\delta_{i\upsilon }\epsilon_{ij\upsilon }$$

where $\gamma_{i\upsilon }$ is related to the coefficients that are associated with the site indicators *i* for feature υ and the parameter $\delta_{i\upsilon }$ describes the multiplicative site effect of the i-th site on feature υ. The procedure for the estimation of the site parameters $\gamma_{i\upsilon }$ and $\delta_{i\upsilon }$ uses Empirical Bayes, as described in [29,31].

For the removal of site effects, three different harmonization procedures were compared: (i) absence of harmonization (no harmonization), which considers as an additional feature the site number identifier expressed as deviations from a baseline site resulting in [N×(P+1)] matrix of features; (ii) removal of site effects using ComBat with age as biological covariate of interest (age covariate); and, (iii) removal of site effects using ComBat without specifying the age as a biological covariate to be preserved (no age covariate). In particular, we applied two version of the ComBat harmonization in order to also test the effect of site removal without taking the chronological age of the subjects into account. Indeed, an interaction between the site variable and the age of the subjects can occur if some sites only include subjects with age in specific ranges and it is therefore important to ensure that the harmonization of the site effect does not affect the age-related biological variability of the dataset. Moreover, for the no harmonization strategy, the information of the site is embedded in a data-driven manner as additional feature and it is a baseline against which the two ComBat harmonization strategies are compared.

### 2.5. Age Prediction

When considering the regression problem of predicting brain ages of N subjects $Y\in R^{N}$ based on the matrix of predicting variables $X\in R^{N\times P}$, we used three different techniques: support vector regression (SVR), random forest, and Lasso regression. Two different metrics were employed to evaluate the regression performance:

- Mean Absolute Error (MAE):
  (4) $$MAE=\frac{1}{N}\sum_{i=1}^{N}\left|y_{i}-\hat{y_{i}}\right|$$
- Coefficient of determination ($R^{2}$):
  (5) $$R^{2}=1-\frac{\sum_{i=1}^{N}{\left(y_{i}-\hat{y_{i}}\right)}^{2}}{\sum_{i=1}^{N}{\left(y_{i}-\bar{y}\right)}^{2}}$$

with N being the sample size, $y_{i}$ the chronological age, $\hat{y_{i}}$ the predicted brain age, and $\bar{y}$ the sample average age.

#### 2.5.1. Support Vector Regression

SVR is a kernel-based machine learning algorithm for regression [41], which estimates the following function:

(6) $$f\left(x\right)=x^{\prime }\beta +b$$

subject to:

(7) $$\forall n:|y_{n}-\left(x_{n}^{\prime }\beta +b\right)|\epsilon$$

In other words, SVR attempts to find a function f(x) that deviates from $y_{n}$ by a value that is no greater than ϵ for each training point $x_{n}$. It can be thought of as a linear regression function in a high dimensional feature space where the input data are mapped via a non-linear function [42]. By using the kernel function, the data can be implicitly mapped into a feature space. In this work, we used a linear kernel and the default parameters (ϵ=0.1) of the SVR implementation in “Caret" R package. Support Vector Machine-Recursive Feature Elimination (SVM-RFE) was adopted to perform feature ranking [43]. This embedded method integrates in a single consistent framework both feature selection and pattern classification: it trains the regression model, computes the ranking of all the features, and removes the features with the smallest ranking criterion. This process is iteratively computed until all of the features have been removed.

#### 2.5.2. Random Forest

Random forest (RF) is an ensemble learner of tree-structured base learners. Each tree individually predicts the target response while the final predictions result from the average of the individual tree predictions [44]. Two source of randomness are recognized in RF: (i) each tree is based on a random subset of the observations, and (ii) each split within each tree is created based on a random subset of mtry candidate predictors. A decision threshold is selected, which partitions the node input samples into two subsets according to a purity measure, so a tree is grown until the nodes have split their inputs into subsets that consist of samples containing just one label. According to random sampling of observations, 36.8% of the observations are not used for any individual tree. These observations are included in the out of the bag (OOB) samples for that tree. The accuracy of RF can be estimated from these OOB samples, as:

(8) $$OOB-MSE=\frac{1}{n}\sum_{i=1}^{n}{\left(y_{i}-\bar{\hat{y_{iO}}}\right)}^{2}$$

where $\bar{\hat{y_{iO}}}$ denotes the average prediction for the *i*th observation from all trees for which this observation has been OOB.

The permutation-based MSE reduction criterion [45] has been adopted to compute RF feature importance. For each variable in each tree, the difference between the OOB-MSE of the permutation of the variable’s out-of-bag data for the tree and the actual OOB-MSE is computed. The MSE reduction according to each predictor for the complete forest is obtained as the average over all trees of these differences. The main idea underlying this algorithm is that if a variable does not have predictive value for the response, the differences between predictions on OOB samples with the actual values of such predictor and predictions with its randomly permuted values are expected to be negligible. We used the “RandomForest” R Package with the default parameter mtry=P/3 and ntree=500.

#### 2.5.3. Lasso

Lasso (Least Absolute Shrinkage and Selection Operator) [46] regression is a regularization method introduced to solve both the multicollinearity problem and overfitting in ordinary least square regression (OLS). This approach introduces a penalty term that controls the complexity of the model with sparsity effect. Indeed, Lasso regression tries to only retain the important features by removing some coefficients of the least significant predictors. The outcome is a subset of the predictors yielding the largest contribution to the regression model, so it is also used as an embedded feature selection method. Lasso minimizes the residual sum of squares (RSS) to find the coefficients of the predictors:

(9) $$RSS=\frac{1}{2}||Y-{\beta X||}_{2}^{2}-{\lambda ||\beta ||}_{1}$$

Tuning the λ parameter is required for the optimization of the accuracy. We used the inner round of each fold of the cross validation to find the best value of λ.

### 2.6. Feature Importance

The output of each regression algorithm is a list of performance metrics; moreover, the ranked features and the number of features $k_{opt}$ to be selected to yield the highest accuracy for each round of cross-validation are returned by the RF and SVR algorithms, while a sparse matrix of weighted coefficients of the regression is output by Lasso. Because, in principle, the features of each subset corresponding to the top $k_{opt}$ elements of each round can be different from one cross-validation round to another, we applied a consensus ranking algorithm to select the most stable features across all of the 100 rounds. Indeed, a feature selection algorithm could be sensitive with respect to changes in the training set, which results in subsets of features not representative of the population under investigation [47]. In order to overcome this limitation, several algorithms have been proposed for quantifying the stability of a ranked or selected list of features with respect to small changes of the training sets drawn from a distribution of samples [48]. Here we adopted:

- robust rank aggregation (RRA) algorithm [49] to combine the multiple base rankers into a final aggregated ranked list for RF and SVR; and,
- frequency-based criterion with a fixed threshold to retain the most frequent features selected in each round of Lasso regression.

The RRA approach computes the position of each item in the final ranking by comparing its position in all of the ranked lists to a non-informative null model of random permutations of the items. A numerical score for each item is then assigned according to the reference beta distributions of order statistics and the Bonferroni correction is applied to compute the P-values and to find the list of statistically significant items. The P-values are then sorted to obtain the final ranking. The algorithm was also extended for accounting partial rankings, i.e., lists, where only the *k* top elements are available.

Because the output produced by Lasso is a sparse matrix of weights, we considered as features that are relevant to the target variable within each round of cross validation those corresponding to non-zero weights. Consequently, the matrix of selected features is a binary matrix, where 1 is found at the (i,j) entry if the *j*th feature has been selected at the round *i*th. Subsequently, we applied a frequency-based criterion by selecting as significantly stable features those that occurred in at least 80% of the rounds. This threshold was chosen while taking into account that the value of 80% is slightly higher than the chance level (i.e., 50%).

### 2.7. Stability Index

In order to verify whether the harmonization strategies affected the most significant age-related regions of interest, we evaluated the overlap between the two sets of selected ROIs that resulted from two different harmonization strategies. In particular, the Jaccard index was used as a measure of the percentage of overlap between two sets, as:

(10) $$J\left(A,B\right)=\frac{\left|A\cap B\right|}{\left|A\cup B\right|}$$

where *A* and *B* are two sets of ROIs. This index represents the proportion of agreement between the two sets of features and it is related to the stability of the features selected with respect to machine learning algorithm [50]. In this case, it represents an index of stability of the most significant anatomical regions for the age prediction with respect to the adopted harmonization strategy. Indeed, since 0≤J≤1, a higher percentage of overlap between the two sets implies that the resulting selected features are stable and invariant with respect to the harmonization algorithm. A permutation test was performed by randomly permuted $10^{4}$ times the selected features between the two sets for determining the statistical significance of their percentage of overlap. Specifically, the permutation test was used to test the null hypothesis that the percentage of overlap between the two sets was given by chance.

### 2.8. Age Models

The predictions of brain age that result from the different cross-validation rounds were averaged for each subject in order to obtain the final age prediction of each regression model and each harmonization strategy. For each of the nine combination (e.g., regression model-harmonization strategy), we evaluated three model fits of chronological age vs. predicted age relation, i.e., linear, quadratic, and cubic fit for each population:

(11) $$\hat{y}=p_{1}y+p_{2}$$

(12) $$\hat{y}=p_{1}y^{2}+p_{2}y+p_{3}$$

(13) $$\hat{y}=p_{1}y^{3}+p_{2}y^{2}+p_{3}y+p_{4}$$

where $\hat{y}$ is the estimated brain age and *y* is the chronological age of the subject. The adjusted explained variance $R^{2}$ was evaluated to select the best-fitting model among the three fits.

To assess the deviation of each model from the ideal age model fit (i.e., $\hat{y}=y$), the normalized area under the fitted curve ($AUC_{\hat{y}}$) of the nine models for both populations were evaluated as:

(14) $$AUC_{\hat{y}}=\frac{2}{{\left(age_{MAX}-age_{MIN}\right)}^{2}}AUC_{model}$$

where $age_{MAX}$ is maximum age value of the sample, $age_{MIN}$ is minimum age value of the sample and $AUC_{model}$ is the area under the model fit. This quantity assumes:

- $AUC_{\hat{y}}=1$ for the ideal model;
- $AUC_{\hat{y}}1$ for an age overestimation prevalence of the model;
- $AUC_{\hat{y}}1$ for an age underestimation prevalence of the model.

Thus, the signed difference between the $AUC_{\hat{y}}$ of the ideal model fit and each of the nine best-fitting models can express the degree of deviation of each model from the ideal one.

## 3. Results

### 3.1. Age Prediction

**Figure 2:**
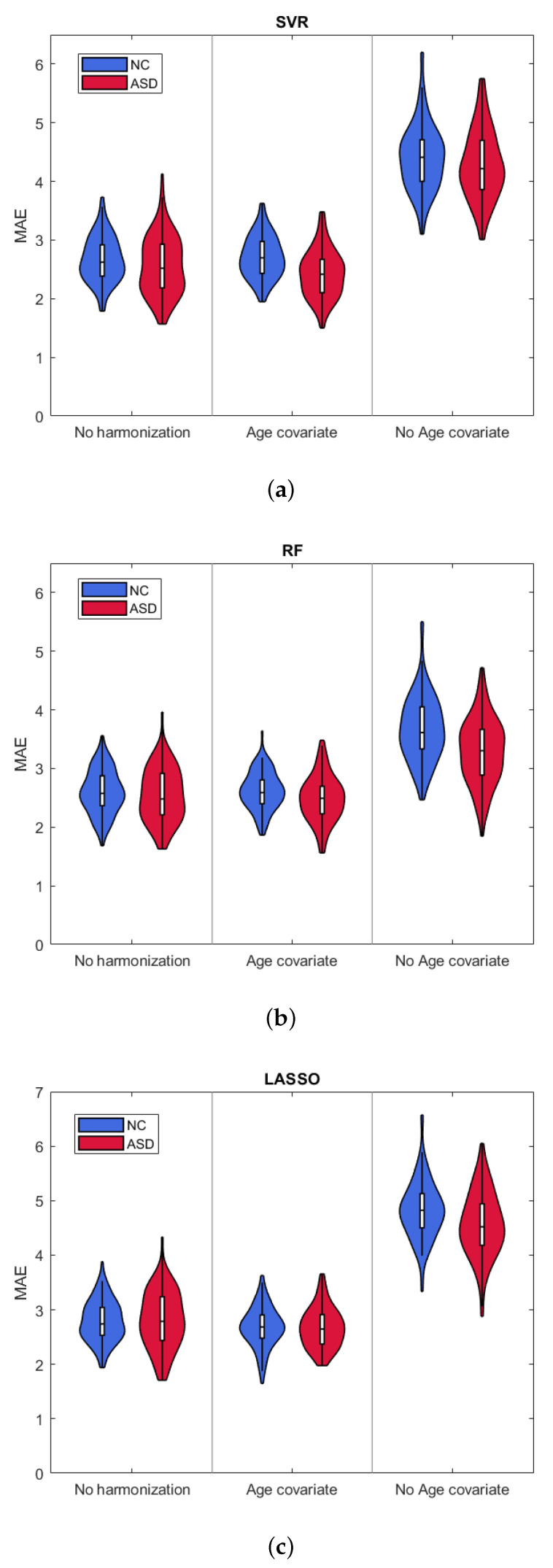
Violin plots of MAE resulting from (**a**) support vector regression (SVR); (**b**) Random forest (RF); and, (**c**) Lasso, for the three harmonization strategies.

We performed the stepwise analysis in order to identify the number of ranked features $k_{opt}$ that minimizes the average MAE values. Table 1 reports the $k_{opt}$ values for the three harmonization strategies and the three regression algorithms for both groups. Figure 2 shows the violin plots of the MAE distributions for the selected $k_{opt}$. Numerical values of the mean and standard deviation of MAE and $R^{2}$ values are also reported in Table 2 and Table 3, respectively. The results clearly show that harmonizing without taking the chronological age of the subjects into account returns the worst performance. Moreover, all of the ML algorithms exhibit comparable results with both not harmonized and age covariate harmonized datasets reporting average MAE2.65, with a slight improvement in the case of the Random Forest algorithm.

### 3.2. Age Models

**Figure 3:**
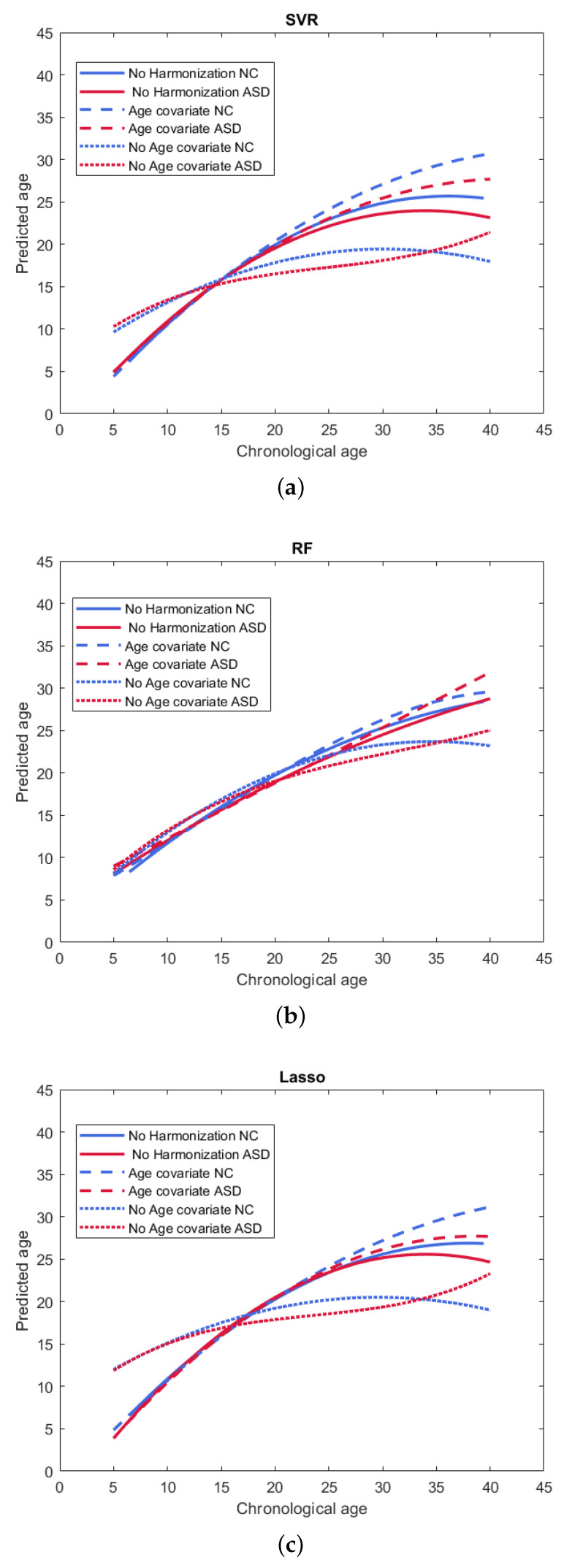
Best-fitting models of chronological age-predicted age resulting from (**a**) SVR; (**b**) RF; (**c**) Lasso, for the three harmonization strategies.

Figure 3 reports the final model fit of the “chronological age–predicted brain age” relation for each of the nine combinations regression model–harmonization strategy. Individual fits with sample points are shown in Figure S1 of Supplementary Information. The adjusted explained variance $R^{2}$ values of the resulting best-fitting models are listed in Table 4. As shown in Figure 3a,c the SVR and Lasso models both achieved similar fits for the two strategies no harmonization and age covariate harmonization. Indeed, these two strategies exhibit the same linear behavior for age 25, while a better quadratic fit is evident in the age covariate harmonization strategy. Moreover, there is always a systematic age underestimation in ASD subjects. The difference between these two harmonization strategies and the subject classes is less evident in the RF fits (see Figure 3b).

The values of the signed difference between the $AUC_{\hat{y}}$ of the ideal model fit and each of the nine best-fitting models are listed in Table 5. For all of the models, a negative value is reported, which suggests a systematic age underestimation. Table 5 also highlights that the RF model yields a smaller deviation from the ideal age model than the other models, which confirms the best performance over the total age range of the sample under investigation.

### 3.3. Feature Importance

**Figure 4:**
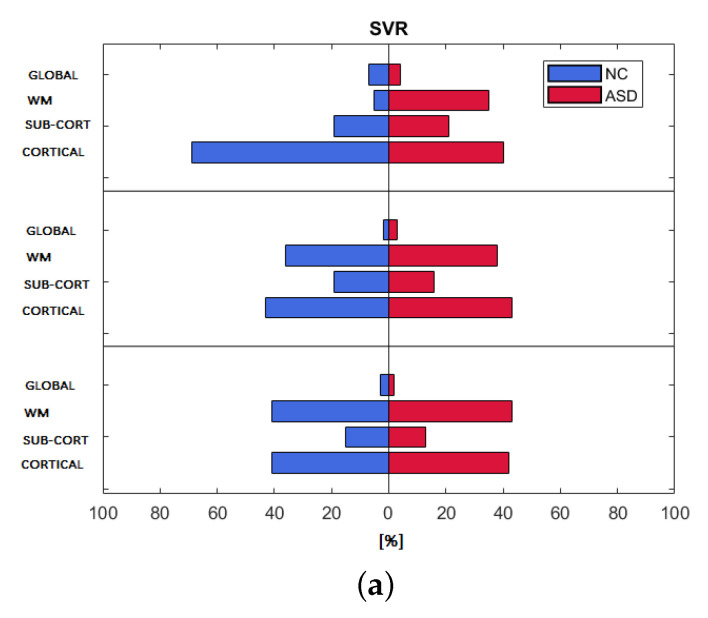

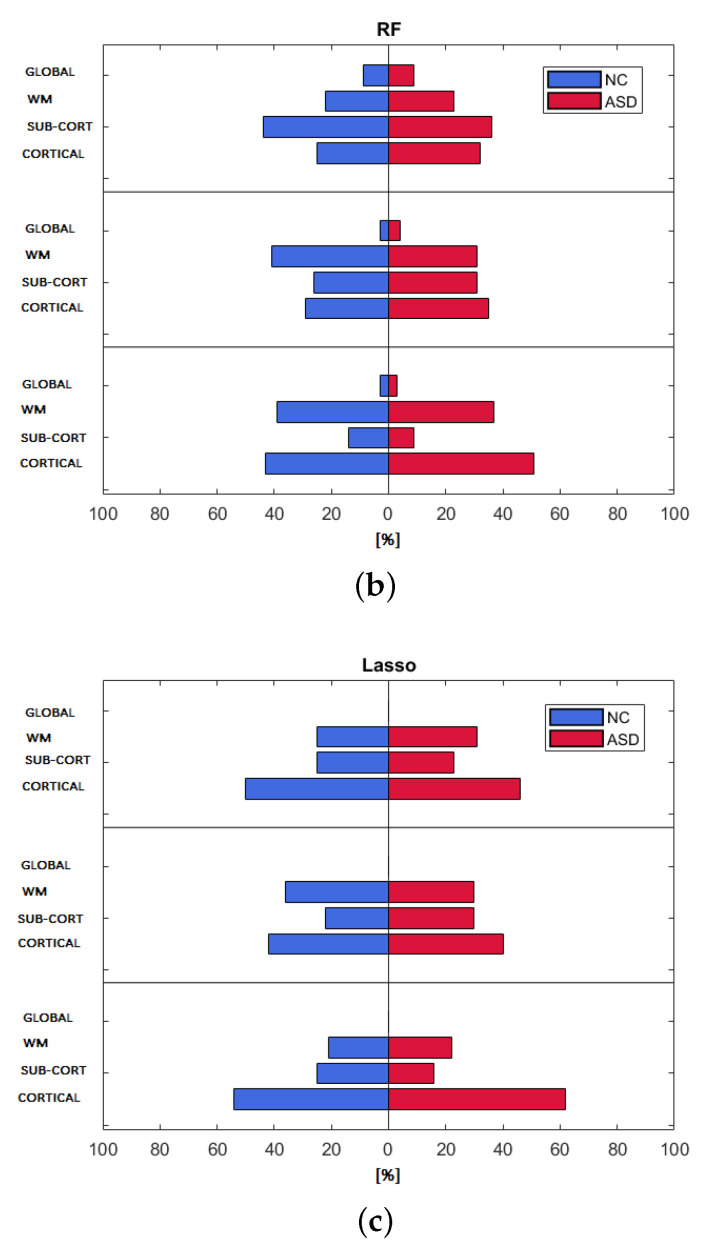
Frequency of occurrence of feature categories resulting from (**a**) SVR; (**b**) RF; (**c**) Lasso, for the three harmonization strategies and both populations.

We computed the relative frequency occurrence of the features’ categories among the $k_{opt}$ features selected by each regression model for the three harmonization strategies in order to show the importance ranking of each category for both populations of subjects. Figure 4 shows the ranking of the four categories. The three regression models exhibit a similar distribution of the importance of the four categories for control subjects and ASD subjects, except for the non-harmonized strategy of the SVR model. Cortical and WM features exhibit a relative frequency of occurrence of approximately 40%, while sub-cortical features appear less than 20% of the times among the features selected by the SVR regression model. For both RF and Lasso models, a greater balancing of the importance of cortical, sub-cortical, and WM features for the age-covariate harmonization occurs, which highlights a frequency of occurrence of about 30%. Cortical features prevail among the $k_{opt}$ features in the Lasso model for all of the harmonization techniques (frequency 50%), while global features are totally missing.

**Figure 5:**
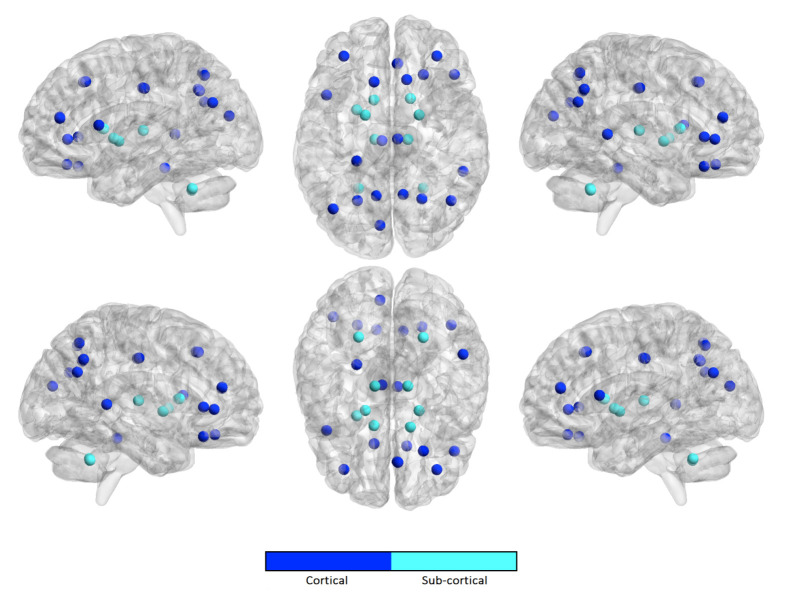
The most significant ROIs for the RF age prediction with no harmonization in NC population.

**Figure 6:**
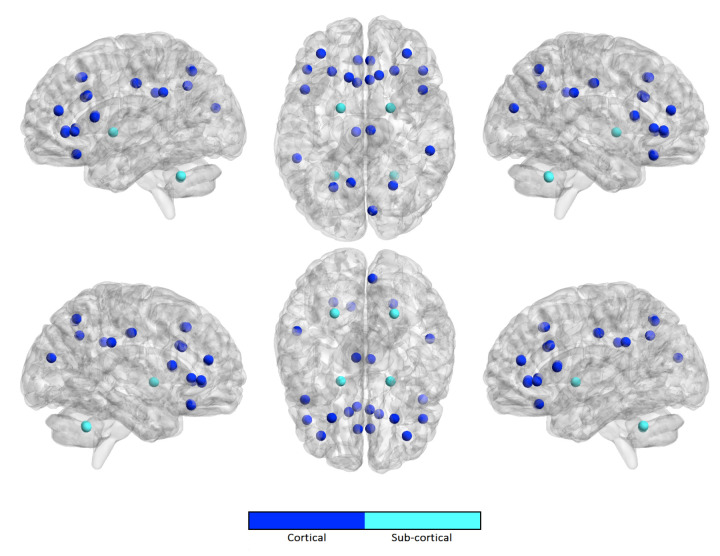
The most significant ROIs for the RF age prediction with age covariate harmonization in NC population.

**Figure 7:**
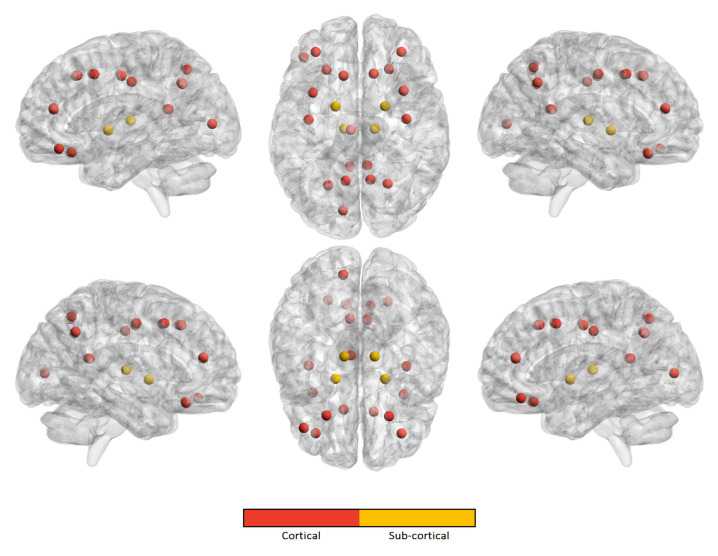
The most significant ROIs for the RF age prediction with no harmonization in ASD population.

**Figure 8:**
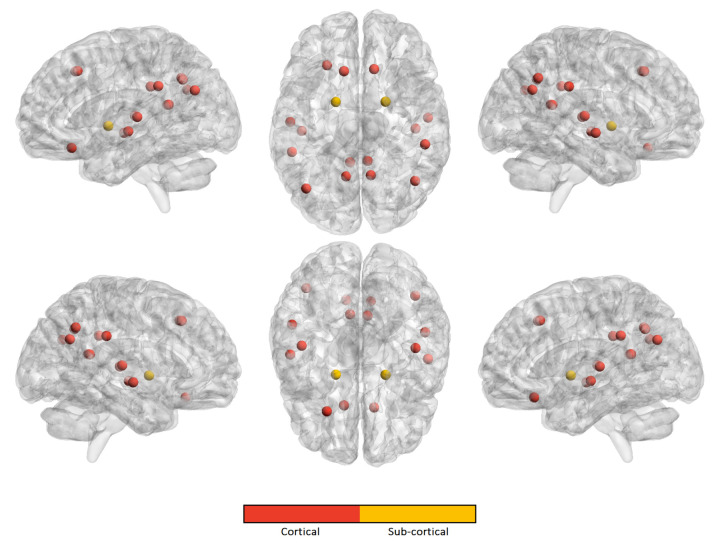
The most significant ROIs for the RF age prediction with age covariate harmonization in ASD population.

Similarly, we computed the occurrence frequency of each ROI across the $k_{opt}$ features to show the most important regions that are involved in age prediction. Figure 5, Figure 6, Figure 7 and Figure 8 only show the most important ROIs for the RF model for the strategies no harmonization and age covariate harmonization, which resulted the most reliable age regression models. Tables S1–S4 of Supplementary Information file also list the same regions with MNI coordinates.

### 3.4. Stability Index

For the random forest classifier, we compared the two sets of selected regions of interest for the two strategies “no harmonization” (i.e., the set A) and “age covariate harmonization” (i.e., the set B) for each of the two groups NC and ASD. The resulting stability index is J(A,B)=0.45, p=0.01 for the NC group and J(A,B)=0.29, p=0.005 for the ASD group, showing a significant greater matching between the selected ROIs of the two harmonization strategies for the control subjects.

## 4. Discussion

In this study, three different regression algorithms were used for age prediction for the three harmonization strategies. The results shown in Figure 2 and summarized in Table 2 and Table 3 indicate that the statistical harmonization without the biological age as coviariate of interest yields the worst performance for both groups, regardless of the regression methods. This finding clearly suggests that, when an interaction occurs between the batch effect (i.e., site) and the biological covariate of interest (i.e., age), performing statistical harmonization while ignoring the covariate leads to confusing effects on the resulting harmonized dataset. On the other hand, it can be observed that the two strategies age-coviariate harmonization and no-harmonization produce almost the same performance for both groups of subjects with an improvement of the mean absolute error with the random forest algorithm. The prediction accuracy that we obtained is in the same range as has been demonstrated by previous studies while using different morphological measures and age prediction methods ($0.6R^{2}0.87$) [22,51,52,53,54,55]. Most of these studies are focused on younger subjects (age20 years) and reported MAE2 [51,52,54,55], while other works showed that the prediction error increases with increasing age with MAE3 in the same age range of our analysis [22,53,56]. Our results seem to confirm such an observation: even though the overall mean prediction errors resulting from random forest age regression for the age covariate harmonization and no harmonization are similar to those reported in literature, all of the models show worse performance for age 25 years. Indeed, a systematic underestimation of the age occurs for subjects older than 25 years, as shown in Figure 3. The analysis of the signed difference between the $AUC_{\hat{y}}$ of the ideal model fit and each of the nine best-fitting models confirms an overall age underestimation of all the regression algorithms as reported in Table 5. However, the deviation from the ideal model is less pronounced for the age covariate harmonization. This analysis also shows greater deviation from the ideal curve for the ASD subjects class than for the NC class for all regression models and harmonization strategies.

The analysis of the importance of the features by category (i.e. cortical, white matter, subcortical, and global), shows that for the strategies of statistical harmonization with age as covariate and for no harmonization, the features related to cortical and white matter statistics, are equally the most important for both classes of subjects, highlighting a greater contribution of cortical areas than subcortical and global structures in the age regression problem. This finding confirms the results of previous work, in which the authors investigated age-related changes of the simultaneous contribution of cortical thickness, regional WM volume, and diffusion characteristics of the brain, concluding that none of the measures are redundant and their integration yields a more complete understanding of brain maturation [57].

For the best performing age regression models, we found that the most informative features for age prediction in control subjects mainly refer to fronto-parietal areas. In particular, the superior frontal cortex is identified in both the harmonized dataset and in the non-harmonized dataset. The precuneus/posterior cingulate (PCC) is also a significant region in both datasets. It is worth noting that several studies confirmed age-related cortical associations of such regions of interest. Both neurodevelopmental and aging analysis revealed distinct chronological structural patterns of different shape metrics in frontal lobe, several parietal regions and cingulate cortex [9,54,55,57,58,59]. In [55], the authors used a multivariate approach to characterize covariation patterns across several cortical and subcortical measures and use them to predict age in a cohort of subjects during the developing stages. Their analysis outlines the role of middle frontal and lateral occipital regions as reliable maturation biomarkers. An accelerated thinning of fronto-parietal regions was also found in [54]. Further, functional and structural connectivity analysis highlighted that the connection between the PCC and mPFC (medial prefrontal cortex), two main hubs of the DMN (default mode network), also plays a fundamental role in neurodevelopment [60,61]. Our study shows that, for the control subjects, a high degree of overlap between the sets of significant cortical regions occurs, highlighting that the statistical harmonization does not introduce major variations with respect to the non-harmonization strategy. This observation is not valid for ASD class where a lower overlap arises between the two sets of features. Indeed in ASD subjects an overlap between the two harmonization techniques is found for the upper and lateral frontal regions and the precuneus, while, among the non-harmonized features, are also included regions of the cingulate and parietal cortex and some regions of the frontal lobe. The effects of age on different subcortical brain volumes has been extensively examined in literature, reporting no relationship between age and the volumes of amygdala and heterogeneous age responses for thalamus, caudate, hippocampus, and cerebellar white and gray matter [62,63,64]. In our analysis, both left and right pallidum appear to be significantly associated with age in both populations for the two harmonization strategies, while regions of striatum are only found among the non-harmonized features of control subjects. It is worth noting that the volume of white and grey matter of the cerebellum is a significant feature for age prediction only for control subjects for both harmonization strategies, while it does not occur among those of ASD subjects. This difference could suggest this feature as a neurodevelopment discriminating factor between the two populations, thus confirming the morphological cerebellum anomalies in ASD reported in several studies [65,66].

In summary, the age-covariate harmonization does not affect the performance of the age prediction models; however, on one hand, it shows less significant effects on predictive features in the control population, but, on the other hand, a greater mismatch of the selected anatomical features between the two strategies was found in ASD subjects, which indicated a more relevant effect of harmonization on the predictive features. Harmonizing a dataset is a fundamental step for multi-site analysis and it is often performed on imaging-derived data before any further analysis. Our results suggest that this process should be embedded into the complete analysis framework in order to assess its effects both on the performance of the algorithms or analysis methods and on the anatomical regions that are relevant to the performed analysis.

## 5. Limitations

Although this study shows important consequences of harmonization on age prediction in a multi-site dataset, it presents some limitations. We estimated the brain age of subjects using different methods and a final index was computed to assess the deviation of each model from the ideal age model fit. In some recent works, various sources of bias in estimating brain age delta have been highlighted, such as the non-Gaussian distribution of subject ages [67,68,69]. The authors in [69] also suggested a procedure for removing bias and increasing the accuracy of delta estimation. Further improvements of the proposed framework will include models for correcting the underestimation of brain aging and removing the resulting dependency of delta on age, as suggested in [69]. In addition, several measures from cognitive testing have been found to be associated with brain age delta; hence, age prediction models could be improved and validated using health or cognitive factors.

Another limitation concerns the data sample: only male participants were included in the analysis due to strong prevalence of male subjects in the ABIDE dataset. Moreover, the dataset does not include multiple MRI measures from the same individual: for further validations of the method, a dataset with multiple measurements should be used to perform the test-retest reliability of the results.

Finally, although cross-validation is commonly used to test the models ability to generalize to an independent dataset while avoiding problems, like overfitting or selection bias [70], independent tests on an external validation set should be required to ensure a better generalization of the age prediction models. Future work will include more datasets to further strengthen the effectiveness of our results.

## 6. Conclusions

In this work, we proposed a novel machine learning framework to evaluate three different harmonization techniques of a cross-sectional dataset for age prediction analysis in two groups of subjects (i.e., controls and ASD). Our analysis aimed to verify the effects of the different harmonization strategies on the final performance and on the set of features that are relevant to the age prediction problem both in the presence and absence of pathology using a stability index to provide a final robust overlap index between the features selected by each ML algorithm for each harmonization strategy.

The results show that the age covariate harmonization and no-harmonization techniques yield comparable results in terms of performance for both groups of subjects, while the statistical harmonization seems to affect the most age-related predictive features. This finding suggests carefully evaluating the effect of the harmonization preprocessing step when planning to predict age on the basis of anatomical measures, especially when analyzing neurodevelopment- or aging-related diseases. The steps performed in the proposed framework can be generalized in order to provide a robust set of relevant features by means of an objective comparison of the outcomes that result from different harmonization strategies to potentially strengthen the relevance of clinical considerations.

## Figures and Tables

**Table 1:** Number of ranked features $k_{opt}$ at which the regression models exhibit the minimum average Mean absolute error (MAE).

Harmonization Technique	NC			ASD		
	SVR	RF	Lasso	SVR	RF	Lasso
No harmonization	400	90	55	150	130	35
Age covariate	1000	100	100	400	50	65
No age covariate	40	30	15	50	20	15

**Table 2:** Mean MAE ± SD resulting from age prediction for each class and each harmonization technique for the three regression algorithms SVR, Random Forest, and Lasso. The best performances are highlighted in gray.

Harmonization Technique	NC			ASD		
	SVR	RF	Lasso	SVR	RF	Lasso
No harmonization	2.66±0.38	2.59±0.37	2.79±0.37	2.56±0.49	2.54±0.45	2.80±0.51
Age covariate	2.70±0.36	2.62±0.32	2.68±0.37	2.46±0.40	2.46±0.39	2.68±0.36
No age covariate	4.40±0.55	3.67±0.54	4.82±0.53	4.28±0.58	3.29±0.55	4.56±0.59

**Table 3:** Mean $R^{2}$± SD resulting from age prediction for each class and each harmonization technique for the three regression algorithms SVR, Random Forest, and Lasso. The best performances are highlighted in gray.

Harmonization Technique	NC			ASD		
	SVR	RF	Lasso	SVR	RF	Lasso
No harmonization	0.76±0.06	0.77±0.06	0.74±0.06	0.72±0.06	0.73±0.08	0.69±0.07
Age covariate	0.77±0.06	0.77±0.06	0.77±0.06	0.76±0.06	0.76±0.07	0.74±0.06
No age covariate	0.27±0.10	0.52±0.11	0.24±0.10	0.30±0.13	0.58±0.10	0.27±0.12

**Table 4:** Best-fitting models of age prediction and adjusted $R^{2}$ for each class and each harmonization technique for the three regression algorithms SVR, Random Forest and Lasso.

Harmonization Technique	Class	SVR	RF	Lasso
No harmonization	NC	$\hat{y}=-0.02y^{2}+1.6y-3.25$	$\hat{y}=-0.01y^{2}+1.16y+1.3$	$\hat{y}=-0.02y^{2}+1.5y-2.6$
		$R^{2}=0.80$	$R^{2}=0.82$	$R^{2}=0.79$
	ASD	$\hat{y}=-0.02y^{2}+1.54y-2.3$	$\hat{y}=-0.006y^{2}+0.88y+3.8$	$\hat{y}=-0.02y^{2}+1.7y-4.2$
		$R^{2}=0.79$	$R^{2}=0.81$	$R^{2}=0.78$
Age covariate	NC	$\hat{y}=-0.01y^{2}+1.4y-2.5$	$\hat{y}=0.01y^{2}+0.65y+4.3$	$\hat{y}=-0.01y^{2}+1.4y-1.8$
		$R^{2}=0.80$	$R^{2}=0.83$	$R^{2}=0.80$
	ASD	$\hat{y}=-0.01y^{2}+1.4y-2.7$	$\hat{y}=0.65y+5.7$	$\hat{y}=-0.02y^{2}+1.6y-3.7$
		$R^{2}=0.83$	$R^{2}=0.80$	$R^{2}=0.79$
No age covariate	NC	$\hat{y}=-0.01y^{2}+0.93y+5.4$	$\hat{y}=-0.01y^{2}+1.22y+2.44$	$\hat{y}=-0.01y^{2}+0.82y+8.2$
		$R^{2}=0.48$	$R^{2}=0.68$	$R^{2}=0.40$
	ASD	$\hat{y}=0.0005y^{3}-0.04y^{2}+1.3y+5.6$	$\hat{y}=-0.0003y^{3}-0.03y^{2}+1.4y+2.4$	$\hat{y}=0.0006y^{3}-0.04y^{2}+1.2y+6.9$
		$R^{2}=0.33$	$R^{2}=0.66$	$R^{2}=0.30$

**Table 5:** Signed difference between the $AUC_{\hat{y}}$ of the ideal model fit and each of the nine best-fitting models for NC and ASD classes.

Harmonization Technique	Class	SVR	RF	Lasso
No harmonization	NC	−0.18	−0.13	−0.15
	ASD	−0.22	−0.15	−0.17
Age covariate	NC	−0.11	−0.10	−0.11
	ASD	−0.16	−0.11	−0.14
No age coviariate	NC	−0.32	−0.18	−0.24
	ASD	−0.34	−0.20	−0.25

## Supplementary Materials

The following are available online at https://www.mdpi.com/2076-3425/10/6/364/s1, Figure S1: Individual fits with sample points of the “chronological age - predicted brain age” relation for each of the nine combinations regression model - harmonization strategy for both population (Control and Autism Spectrum Disorder), Table S1: most important ROIs with MNI coordinates for the RF model for the age covariate harmonization in ASD population, Table S2: most important ROIs with MNI coordinates for the RF model for the age covariate harmonization in NC population, Table S3: most important ROIs with MNI coordinates for the RF model for the no harmonization strategy in ASD population, Table S4: most important ROIs with MNI coordinates for the RF model for the no harmonization strategy in NC population.
