# Supplementary material for: Extensive Evaluation of Morphological Statistical Harmonization for Brain Age Prediction
Source: Brain Sci. 2020 Jun 11;10(6):364. doi: 10.3390/brainsci10060364 (PMC7349402; doi:10.3390/brainsci10060364)
Supplement: Supplementary file 1 [file brainsci-10-00364-s001.pdf]

Supplementary Information

Extensive Evaluation of Morphological Statistical Harmonization for Brain Age Prediction

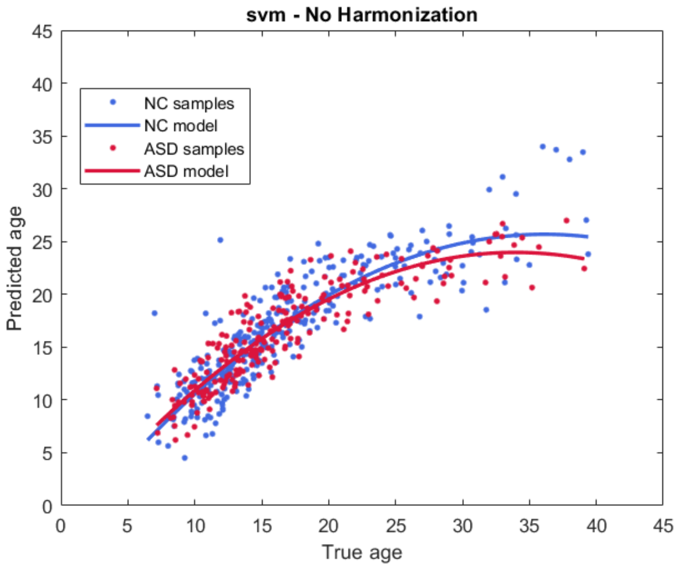

(a)

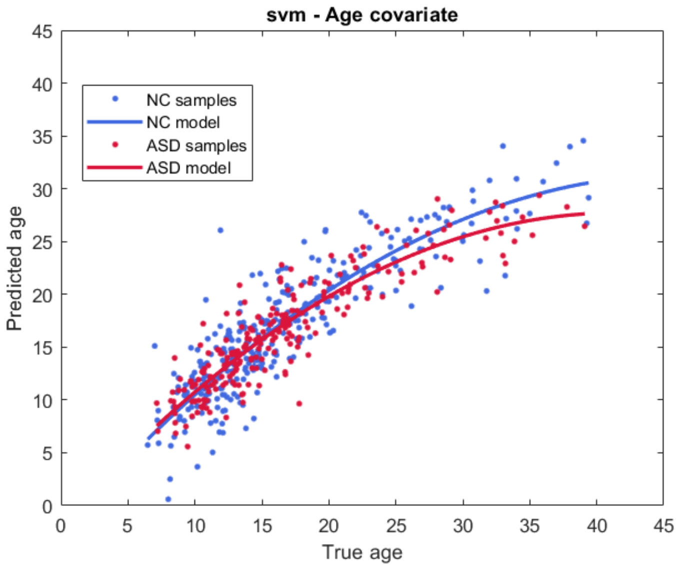

(b)

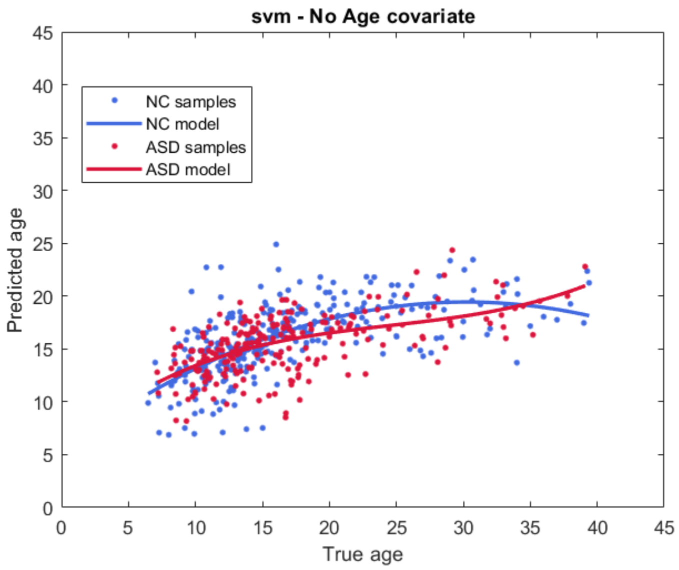
svm - No Age covariate
NC samples
NC model
ASD samples
ASD model
Predicted age
True age
0
5
10
15
20
25
30
35
40
45

(c)

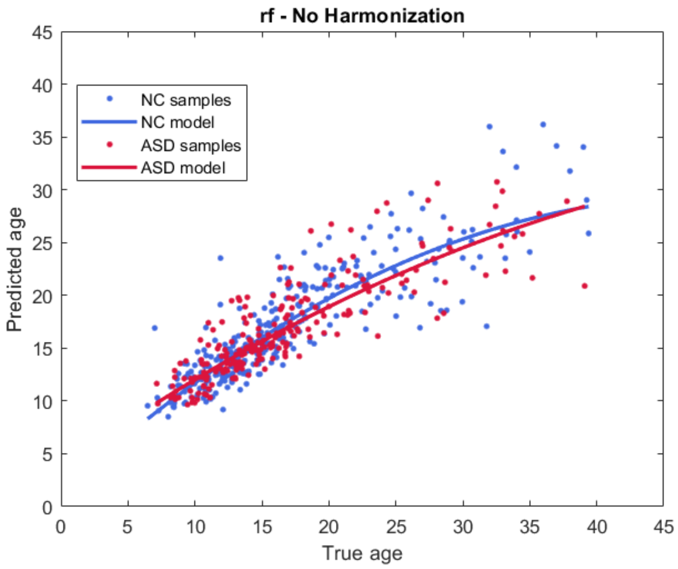
rf - No Harmonization
NC samples
NC model
ASD samples
ASD model
Predicted age
True age
0
5
10
15
20
25
30
35
40
45

(d)

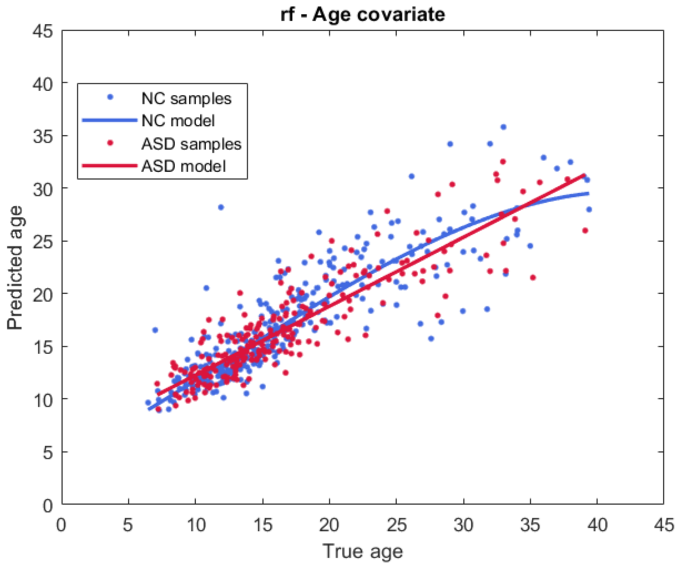
rf - Age covariate
NC samples
NC model
ASD samples
ASD model
Predicted age
True age
0
5
10
15
20
25
30
35
40
45

(e)

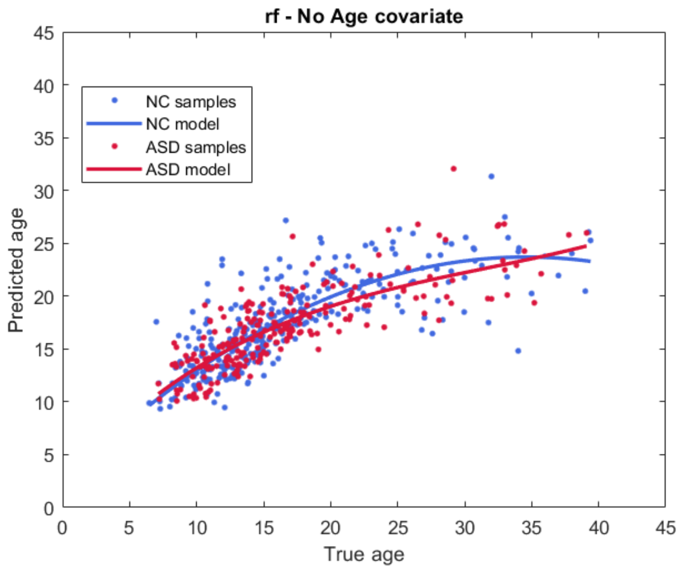
rf - No Age covariate
NC samples
NC model
ASD samples
ASD model
Predicted age
True age
0
5
10
15
20
25
30
35
40
45

(f)

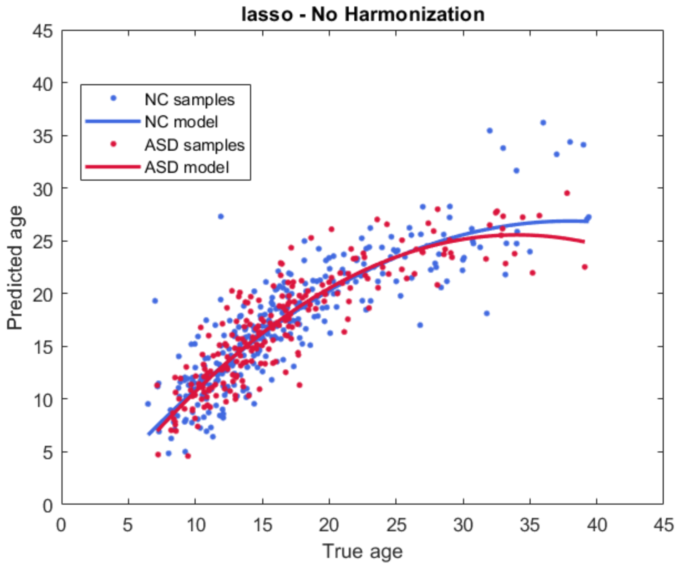
lasso - No Harmonization
NC samples
NC model
ASD samples
ASD model
Predicted age
True age
0
5
10
15
20
25
30
35
40
45

(g)

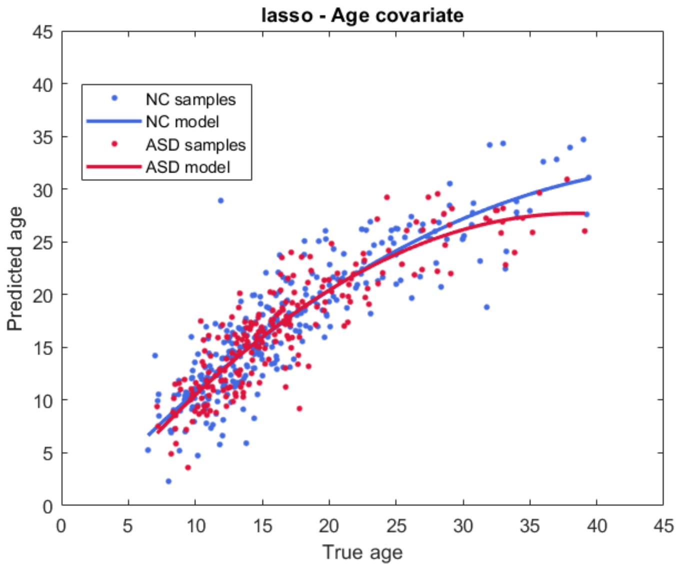
lasso - Age covariate
NC samples
NC model
ASD samples
ASD model
Predicted age
True age
0
5
10
15
20
25
30
35
40
45

(h)

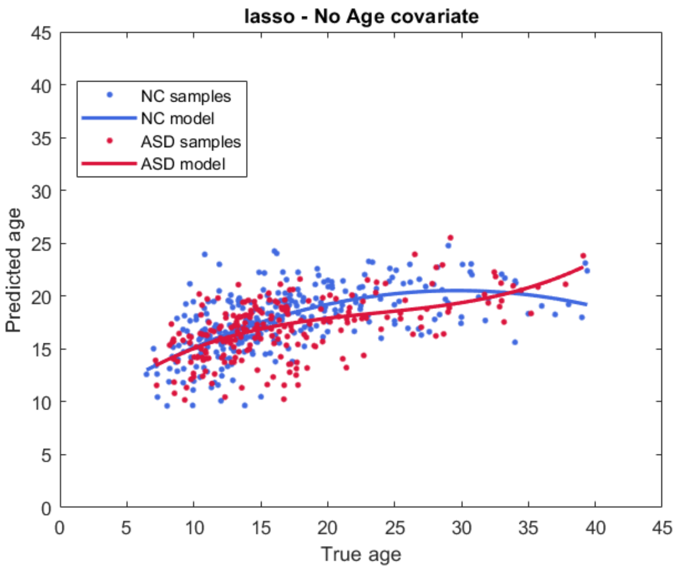

(i)

SI-Figure 1: Individual fits with sample points of the "chronological age - predicted brain age" relation for each of the nine combinations regression model - harmonization strategy for both population (Control and Autism Spectrum Disorder) : (a) SVR with no harmonization, (b) SVR with age-covariate harmonization, (c) SVR with no age-covariate harmonization, (d) RF with no harmonization, (e) RF with age-covariate harmonization, (f) RF with no age-covariate harmonization, (g) Lasso with no harmonization, (h) Lasso with age-covariate harmonization, (i) Lasso with no age-covariate harmonization.

MNI coordinates			ROI
-40.93	-67.71	28.12	l.inferiorparietal
-6.62	-47.24	16.96	l.isthmuscingulate
-24.78	28.71	-16.96	l.lateralorbitofrontal
-9.69	-58.23	36.66	l.precuneus
-11.37	24.09	43.37	l.superiorfrontal
-53.40	-15.66	-4.00	l.superiortemporal
-52.06	-39.12	31.48	l.supramarginal
-44.47	-22.67	7.33	l.transversetemporal
-17.75	-0.03	0.21	Left-pallidum
44.34	-61.78	28.63	r.inferiorparietal
7.09	-46.16	16.74	r.isthmuscingulate
9.63	-57.31	37.84	r.precuneus
12.19	25.69	43.07	r.superiorfrontal
54.38	-12.26	-5.11	r.superiortemporal
52.10	-33.12	31.19	r.supramarginal
44.65	-20.79	8.16	r.transversetemporal
21.2	0.18	0.23	Right-pallidum

SI-Table 1: most important ROIs with MNI coordinates for the RF model for the age covariate harmonization in ASD population.

MNI coordinates			ROI
-5.03	20.08	28.99	l.caudalanteriorcingulate
-24.78	28.71	-16.96	l.lateralorbitofrontal
-45.74	14.55	11.85	l.parsopercularis
-44.01	30.26	0.80	l.parstriangularis
-5.70	-18.39	38.47	l.posteriorcingulate
-9.69	-58.23	36.66	l.precuneus
-4.38	37.52	-0.21	l.rostralanteriorcingulate
-33.21	42.71	16.83	l.rostralmiddlefrontal
-11.37	24.09	43.37	l.superiorfrontal
-23.40	-61.78	47.82	l.superiorparietal
-52.06	-39.12	31.48	l.supramarginal
-17.75	-0.03	0.21	Left-pallidum
-22.60	-52.98	-33.95	Left-cerebellum
5.01	22.25	27.63	r.caudalanteriorcingulate
7.16	-80.09	19.16	r.cuneus
24.23	29.34	-17.99	r.lateralorbitofrontal
46.52	14.24	13.37	r.parsopercularis
46.62	29.45	3.34	r.parstriangularis
5.68	-17.19	38.85	r.posteriorcingulate
5.36	37.10	1.67	r.rostralanteriorcingulate
33.96	42.83	17.68	r.rostralmiddlefrontal
12.19	25.69	43.07	r.superiorfrontal
23.19	-60.47	49.69	r.superiorparietal
52.10	-33.12	31.19	r.supramarginal
21.2	0.18	0.23	Right-pallidum
24.15	-52.73	-35.14	Right-cerebellum

SI-Table 2: most important ROIs with MNI coordinates for the RF model for the age covariate harmonization in NC population.

MNI coordinates			ROI
-35.52	10.80	44.19	l.caudalmiddlefrontal
-6.62	-47.24	16.96	l.isthmuscingulate
-24.78	28.71	-16.96	l.lateralorbitofrontal
-42.51	38.55	-14.10	l.parsorbitalis
-11.76	-81.49	5.36	l.pericalcarine
-5.70	-18.39	38.47	l.posteriorcingulate
-38.79	-10.41	42.97	l.precentral
-9.69	-58.23	36.66	l.precuneus
-33.21	42.71	16.83	l.rostralmiddlefrontal
-11.37	24.09	43.37	l.superiorfrontal
-23.40	-61.78	47.82	l.superiorparietal
-17.75	-0.03	0.21	Left-pallidum
-10.85	-17.56	7.98	Left-thalamus
35.66	12.29	44.47	r.caudalmiddlefrontal
7.09	-46.16	16.74	r.isthmuscingulate
24.23	29.34	-17.99	r.lateralorbitofrontal
37.89	-9.81	44.54	r.precentral
9.63	-57.31	37.84	r.precuneus
33.96	42.83	17.68	r.rostralmiddlefrontal
12.19	25.69	43.07	r.superiorfrontal
23.19	-60.47	49.69	r.superiorparietal
21.2	0.18	0.23	Right-pallidum
13	-17.55	8.09	Right-thalamus

SI-Table 3: most important ROIs with MNI coordinates for the RF model for the no harmonization strategy in ASD population.

MNI coordinates			ROI
-7.12	-79.63	18.51	l.cuneus
-40.93	-67.71	28.12	l.inferiorparietal
-23.90	-33.14	-19.24	l.parahippocampal
-45.74	14.55	11.85	l.parsopercularis
-5.70	-18.39	38.47	l.posteriorcingulate
-9.69	-58.23	36.66	l.precuneus
-33.21	42.71	16.83	l.rostralmiddlefrontal
-11.37	24.09	43.37	l.superiorfrontal
-23.40	-61.78	47.82	l.superiorparietal
-11.46	11.00	9.24	Left-caudate
-17.75	-0.03	0.21	Left-pallidum
-10.85	-17.56	7.98	Left-thalamus
-23.91	3.86	2.4	Left-putamen
-22.60	-52.98	-33.95	Left-cerebellum
52.97	-40.55	5.30	r.bankssts
44.34	-61.78	28.63	r.inferiorparietal
24.23	29.34	-17.99	r.lateralorbitofrontal
5.85	37.56	-16.58	r.medialorbitofrontal
46.62	29.45	3.34	r.parstriangularis
5.68	-17.19	38.85	r.posteriorcingulate
9.63	-57.31	37.84	r.precuneus
12.19	25.69	43.07	r.superiorfrontal
23.19	-60.47	49.69	r.superiorparietal
5.36	37.10	1.67	r.rostralanteriorcingulate
33.96	42.83	17.68	r.rostralmiddlefrontal
14.84	12.07	9.42	Right-caudate
21.2	0.18	0.23	Right-pallidum
13	-17.55	8.09	Right-thalamus
24.15	-52.73	-35.14	Right-cerebellum

SI-Table 4: most important ROIs with MNI coordinates for the RF model for the no harmonization strategy in NC population.
